# Morphometric measurement of the proximal tibia to design the tibial component of total knee arthroplasty for the Thai population

**DOI:** 10.1186/s40634-021-00429-9

**Published:** 2021-12-20

**Authors:** Chotchuang Phombut, Supakit Rooppakhun, Bura Sindhupakorn

**Affiliations:** 1 School of Mechanical Engineering, Suranaree University of Technology, Nakhon Ratchasima, 30000 Thailand; 2 School of Orthopedics, Suranaree University of Technology, Nakhon Ratchasima, 30000 Thailand

**Keywords:** Proximal tibia, Morphometric, Thais, Tibial component, Reverse engineering

## Abstract

**Purpose:**

This study evaluates the morphology of the Thai proximal tibia based on three-dimensional (3D) models to design the tibial component.

**Methods:**

The 3D models of 480 tibias were created using reverse engineering techniques from computed tomography imaging data obtained from 240 volunteers (120 males, 120 females; range 20–50 years). Based on 3D measurements, a digital ruler was used to measure the distance between the triangular points of the models. The morphometric parameters consisted of mediolateral length (ML), anteroposterior width (AP), medial anteroposterior width (MAP), lateral anteroposterior width (LAP), central to a medial length (CM), central to a lateral length (CL), medial anterior radius (MAR), lateral anterior radius (LAR), and tibial aspect ratio (AR). An independent t-test was performed for gender differences, and K-means clustering was used to find the optimum sizes of the tibial component with a correlation between ML length and AP width in Thai people.

**Results:**

The average morphometric parameters of Thai proximal tibia, namely ML, AP, MAP, LAP, CM, and CL, were as follows: 72.52 ± 5.94 mm, 46.36 ± 3.84 mm, 49.22 ± 3.62 mm, 43.59 ± 4.05 mm, 14.29 ± 2.72 mm, and 15.28 ± 2.99 mm, respectively. The average of MAR, LAR, and AR was 24.43 ± 2.11 mm, 21.52 ± 2.00 mm, and 1.57 ± 0.08, respectively. All morphometric parameters in males were significantly higher than those of females. There was a difference between the Thai proximal tibia and other nationalities and a mismatch between the size of the commercial tibial component and the Thai knee. Using K-means clustering analysis, the recommended number of ML and AP is seven sizes for the practical design of tibial components to cover the Thai anatomy.

**Conclusion:**

The design of the tibial component should be recommended to cover the anatomy of the Thai population. These data provide essential information for the specific design of Thai knee prostheses.

## Introduction

*Total knee arthroplasty* (TKA) is the preferred surgical procedure for severe knee osteoarthritis treatment, relieving pain and restoring function [18, 29]. A successful outcome in TKA is dependent on precise bone cutting, appropriate soft tissue balancing, and maximum tibial bone coverage with suitable implant size [6, 18]. The correct size of the implant would reduce the stress applied between bone and implant interface [2, 17, 18]. Preferable compatibility between the implant and the resected surface of the bone is an essential factor for long-term survivorship in TKA [6]. In TKA, a tibial component is more highly susceptible to complications than a femoral component [4]. If a tibial component does not fit the resected surface of the proximal tibia, an orthopedic surgeon may be required to select either an oversized or undersized member. After TKA, a significant tibial overhanging may cause soft tissue irritation and knee pain, particularly on the medial side [3, 26]. Conversely, an underhanging tibial component has the potential to increase tibial bone resorption, which is one of the causes of aseptic loosening [12]. Furthermore, tibial component subsidence causes misalignment and increases wear instability [35, 38]. Therefore, morphometric measurements of the proximal tibia are essential for designing and manufacturing tibial components.

Generally, two methods were used for the morphometric study of knee joints: direct and indirect. Using rulers and Vernier calipers, the direct method measured dry bone, cadaveric, and intraoperative determination. The indirect method, which included a computed tomography scan (CT) image, magnetic resonance imaging (MRI), and three-dimensional (3D) models, was also used. The advantage is that the investigation was performed digitally, which was non-destructive to the specimen. Currently, 3D models are widely used for morphometric studies of the knee joint [13, 24, 25, 32, 40]. The 3D models have been created using a reverse engineering technique, which involves digitizing and reconstructing the actual object using virtual models. The digitization phase consists of data collected from physical objects such as a sample part or prototype using various scanners. Scanning data are obtained in 3D coordinates or images representing the object’s surface. The data are transferred to Computer-Aided Reverse Engineering (CARE) software during the reconstruction phase, which reconstructs the three-dimensional model with the surface represented as a polygonal mesh [13, 25, 32, 34, 40]. Based on 3D models, the digital ruler is used for measurement and assessment, which is sufficiently precise and efficient. Furthermore, the models can be customized to cut, translate, and rotate [11].

The conventional parameters consisting of mediolateral length, anteroposterior width, and the tibia aspect ratio were reported [8, 14, 37, 39]. In a previous study, the anthropometric measurement of knee joints in the Thai population using MRI was reported [5]. However, morphometric parameters of the Thai proximal tibia may be inadequate for implementation in improving knee implant design. The dimension of the proximal tibia is an essential consideration in the tibial component design of total knee arthroplasty. Maximizing the surface area of coverage between the resected surface of the bone and the implant component is one approach to extending the life of knee prosthesis [17]. Therefore, this study aims to evaluate fundamental morphometric parameters of the Thai proximal tibia based on a 3D model to be a cost-effective, quick, and non-invasive method. The morphometric data of the proximal tibia are compared to the tibial component available in commercial use. The mediolateral length and anteroposterior width data are typically used to classify the numbers of tibial component sizing. The proper size of the tibial component is performed by K-means clustering analysis and the elbow technique [27]. This study hypothesized that the morphometric data from the left and right tibias are not different. In addition, there are no different morphometric parameters between males and females, and the Thai proximal tibia has no differences from other nationalities.

## Material and methods

### Data acquisition and 3D reconstruction

**Fig. 1:**
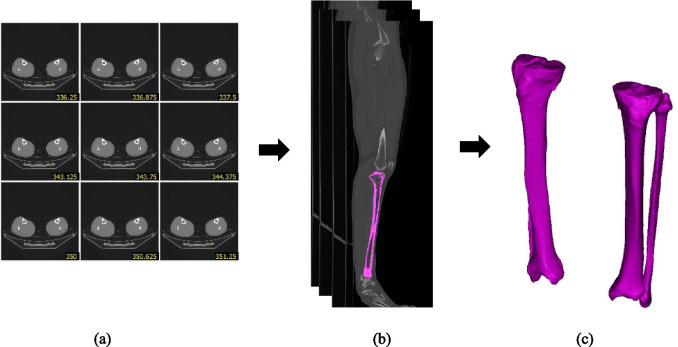
3D model reconstruction of the tibia bone. **a** a series of raw CT images, **b** Segmentation, and **c** 3D tibia model

Suranaree University of Technology’s ethics committee for human subjects research had approved this study (EC59-60). Participants were selected from Thai volunteers ranging in age from 20 to 50 years. 480 Thai tibiae were collected from 240 volunteers (120 males and 120 females). Everyone participating in the study was informed and signed a consent form. Each participant was chosen based on the following criteria: no clinical history of knee arthritis, normal lower limb appearance, and regular alignment. Exclusion criteria included being underage, being suspected of being pregnant due to a loss of menstruation for more than 1 month, as well as refusing to undergo CT. The participants were placed in a supine position with their feet in a neutral position using a 64-slice spiral computed tomography scanner (Optima CT660, GE Healthcare, Chicago, IL, USA). The CT protocol was performed as follows: 120 kVA with an automatic value in the range of 50 to 320 mA, a dose reduction of 20%, and a slice thickness of 2.5 mm with a reconstruction of 0.625 mm in a scan length of 30 to 90 cm. The CT images were then imported into MIMICS Research 20.0 software (Materialise N.V., Leuven, Belgium) for segmentation. The bone-specific present from the Hounsfield unit range was employed for automatic bone segmentation (226 - 3071 HU). After the segmentation process was complete, an integrated function calculated the 3D model from the CT images was exported in stereolithography (STL) format (Fig. 1).

### Tibial resection model and measurements

**Fig. 2:**
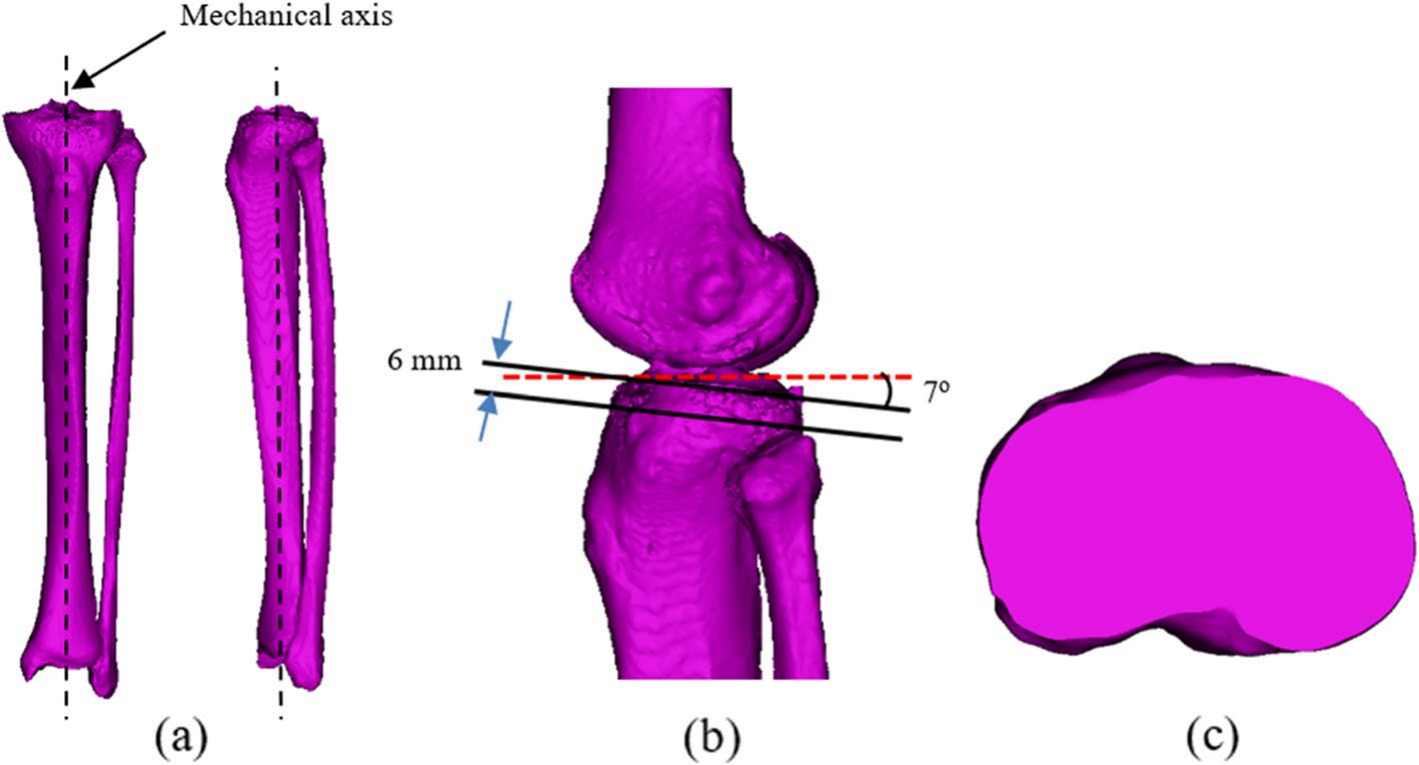
3D model of the tibia bone. **a** Tibial mechanical axis, **b** Position of the resected plane of the tibia, and **c** Resected surface of the tibia

Before measurement, the STL files of proximal models were exported to Materialise 3-Matic Research 12.0 (Materialise N.V., Leuven, Belgium) for adjustment. As shown in Fig. 2, the proximal tibia was measured at the level of tibial resection, 6 mm below the lateral tibial plateau and perpendicular to the tibial mechanical axis with a 7-degree posterior slope [8]. The tibial mechanical axis was created by connecting the center of the proximal tibia and the ankle [28].

**Fig. 3:**
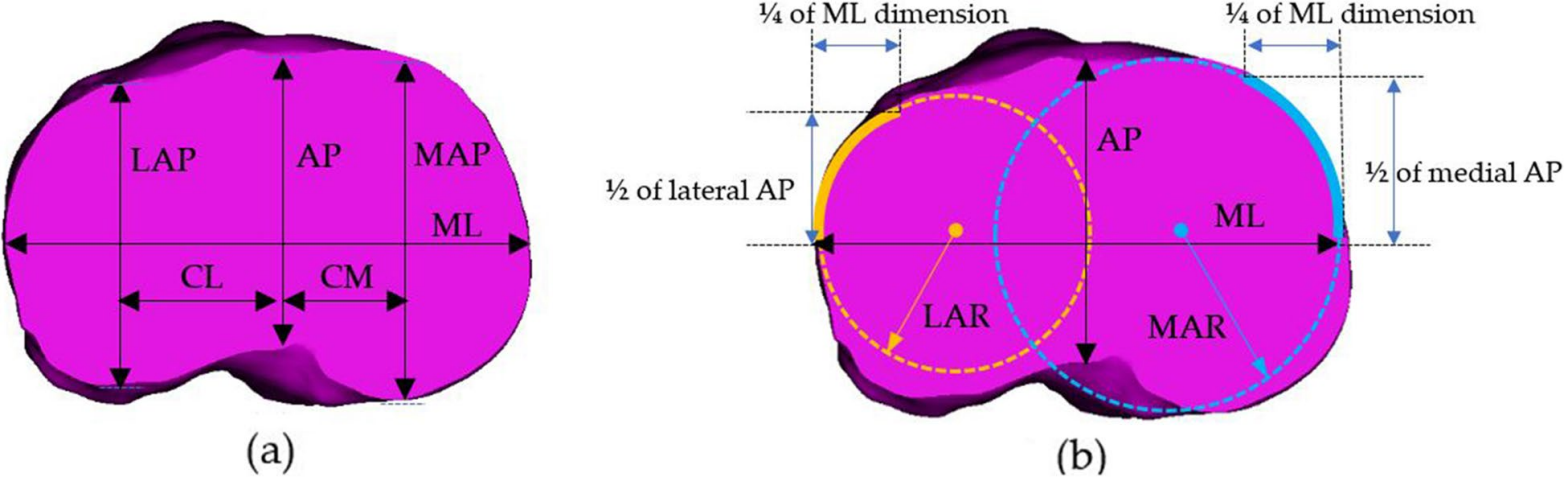
The morphometric parameters of the proximal tibia. **a** linear measurement and **b** curvature measurement

Figure 3 shows the morphometric measurement consisting of anteroposterior width, mediolateral length, medial and lateral anteroposterior width, central to medial length, central to lateral length, medial and lateral anterior radius, and tibial aspect ratio. The medial curvature was defined as the resection contour from the medial one-fourth of the mediolateral length for curvature measurement. The anterior curve of the medial side was defined as the anterior one-half of medial anteroposterior width. The radius of the least square best-fit circle to the anterior medial curve was then determined as the radius of the medial anterior radius. The lateral anterior radius was defined in the same way [9]. Before any measuring and instrument or assessment tool for research or clinic applications are used, reliability must be established. The intra-class correlation coefficient (ICC) is a commonly used statistic to evaluate reliability, such as intra- and inter-observer agreements [16]. Two observers measured morphometric parameters on 20 random tibias in three replicates, and one observer measured the same tibia twice with a two-week interval. The morphometric parameters of the proximal tibia, as shown in Table 1, were abbreviated and defined as follows: three lengths, three widths, two radii, and one ratio.

**Table 1:** Abbreviation and definition of proximal tibial morphometric parameters

Abbreviation	Measurement	Definition
ML	Mediolateral length	The longest mediolateral line of the proximal cut tibial surface, parallel and collinear to the surgical epicondylar axis of the femur
AP	Anteroposterior width	The distance of a perpendicular line is drawn through the midpoint of the ML
MAP	Medial anteroposterior width	The distance of a parallel line to the AP line and passed through the extreme posterior point to the extreme anterior point of the medial tibial condyle
LAP	Lateral anteroposterior width	The distance of a parallel line to the AP line and passed through the extreme posterior point to the extreme anterior point of the lateral tibial condyle
CM	Central to a medial length	The distances of the MAP lines to the AP line.
CL	Central to a lateral length	The distances of the LAP lines to the AP line.
MAR	Medial anterior radius	The least-squares best-fit circle creates the radius-to-anterior medial profile
LAR	Lateral anterior radius	The least-squares best-fit circle creates the radius-to-anterior lateral profile
AR	Aspect ratio	Tibial aspect ratio: ML length divided by AP width

### Statistical analysis

The data were presented, including the mean and standard deviation (SD). A paired t-test was then used to evaluate the differences in the variables between the sides. An independent t-test was performed to compare mean differences between genders. Pearson’s correlation coefficient (r) was calculated to determine the relationships between the morphometric parameters. A statistically significant difference was defined by a *p*-value less than 0.05. Furthermore, the K-means clustering analysis and the elbow method were used to determine proper tibial component sizes. The statistical software SPSS (SPSS Inc., Chicago, IL, USA) analyzed the measured data.

## Results

### Reliability analyses

Accordingly, intra-class correlation coefficient analysis (Table 2), all parameters were found to be highly reliable in the 0.88 to 0.99 range, according to intra-rater reliability. Likewise, the inter-rater reliability value was between 0.80 and 0.97. The high value of ICC indicated that the measurement was highly reproducible [22].

**Table 2:** The value of the intra-class correlation coefficient for various morphometric parameters

Parameters	Intra-rater	Inter-rater
ML	0.98	0.97
AP	0.95	0.94
MAP	0.95	0.89
LAP	0.98	0.81
CM	0.88	0.94
CL	0.89	0.85
MAR	0.99	0.80
LAR	0.98	0.80

### Comparison of morphometric parameters of the proximal tibia between sides

Table 3 shows the results of the statistical comparison of the measurements of the left and right tibia. There were no statistically significant differences between these measurements. This finding was similar to the previous research, namely the morphometric study of the femur and ankle [6, 19]. Thus, the morphometric or anthropometric studies of a femur, tibia, and ankle could be performed either left or right.

**Table 3:** Symmetry analysis of the proximal tibia parameters in mean ± SD (mm)

Parameters	Right (***n*** = 240)	Left (***n*** = 240)	***p***-value
ML	72.49 ± 5.90	72.55 ± 5.99	0.444
AP	46.29 ± 3.81	46.42 ± 3.87	0.062
MAP	49.21 ± 3.64	49.22 ± 3.60	0.886
LAP	43.58 ± 4.04	43.60 ± 4.06	0.664
CM	14.27 ± 2.70	14.30 ± 2.74	0.662
CL	15.30 ± 2.97	15.24 ± 3.00	0.421
MAR	24.40 ± 2.13	24.45 ± 2.10	0.058
LAR	21.48 ± 2.01	21.54 ± 1.99	0.077
AR	1.57 ± 0.07	1.56 ± 0.08	0.308

### Summary of morphometric measurement of the proximal tibia

Table 4 summarizes the morphometric parameters of the proximal tibia in terms of mean ± SD (mm). The results exhibited a significant difference between the genders based on the available data. The male group had higher morphometric parameters than the female group (*p* < 0.05). However, the *p*-value of AR was 0.012, indicating a statistically significant difference. However, the aspect ratio of Thai tibial may range from 1.55 to 1.57.

**Table 4:** Summary of Thai proximal tibia morphology measurements in mean SD (mm)

Parameters	Total (***n*** = 480)	Male (***n*** = 240)	Female (***n*** = 240)	***p***-value
ML	72.52 ± 5.94	77.52 ± 3.22	67.51 ± 3.17	< 0.0001
AP	46.36 ± 3.84	49.26 ± 2.49	43.45 ± 2.53	< 0.0001
MAP	49.22 ± 3.62	51.79 ± 2.64	46.65 ± 2.44	< 0.0001
LAP	43.59 ± 4.05	46.73 ± 2.73	40.45 ± 2.37	< 0.0001
CM	14.29 ± 2.72	15.71 ± 2.65	12.87 ± 1.92	< 0.0001
CL	15.28 ± 2.99	16.35 ± 3.11	14.20 ± 2.43	< 0.0001
MAR	24.43 ± 2.11	26.00 ± 1.41	22.86 ± 1.43	< 0.0001
LAR	21.52 ± 2.00	23.00 ± 1.38	20.03 ± 1.29	< 0.0001
AR	1.57 ± 0.08	1.57 ± 0.07	1.55 ± 0.09	0.012

### Correlation and linear regression analyses

The first ten of the linear regression and high correlation coefficients of the morphology of Thai proximal tibia are shown in Table 5. The relationship between ML and LAP had the highest correlation coefficient value: 0.86. However, another parameter relation had strong coefficients of correlation of more than 0.7 observed. For the rest of the parameter pairs, the r-value falls within a range between 0.17 and 0.74. Furthermore, high and robust parameter correlation pairs were shown as linear regression equations to be helpful in predicting other parameters.

**Table 5:** The equations of morphometric parameters’ high pairwise correlation for Thais

Parameters	Linear regression equation	Correlation coefficient (r)
ML vs. LAP	LAP = 1.21 + 0.58ML	0.859
LAP vs. AP	LAP = 2.65 + 0.88AP	0.838
ML vs. MAP	MAP = 13.10 + 0.50ML	0.818
MAP vs. LAP	MAP = 17.51 + 0.73LAP	0.814
MAR vs. LAR	LAR = 2.74 + 0.77MAR	0.812
ML vs. AP	AP = 0.83 + 0.52ML	0.811
MAP vs. AP	AP = 4.42 + 0.85MAP	0.802
ML vs. LAR	LAR = 1.92 + 0.27ML	0.802
MAR vs. ML	MAR = 5.00 + 0.27ML	0.753
MAR vs. MAP	MAR = 3.02 + 0.44MAP	0.744

### Comparison of morphometric measurement with other populations

Using a two-sample t-test, Table 6 shows the result of comparison morphometric parameters with various populations, including Indians, Koreans, Americans, Japanese, Chinese, Iranians, and Turkish. This study found that the ML of Thai differed from most ethnic groups, except for Iranians. However, the Thai ML was similar to Asians but smaller than Americans and Caucasians. There were significant differences in AP of Thai to Koreans, Japanese, Chinese, and Turkish, but slightly lower than Japanese and Chinese. The MAP of Thai was different from other populations and greater than Indians and Koreans. In addition, the LAP differed from other populations except for Turkish. The average of AR in the Thais was significantly higher than Caucasian, which was close to Koreans and Chinese but smaller than Iranians and Turkish.

**Table 6:** Comparison of the morphometric parameters with other nationalities in mean ± SD (mm)

Studies	Parameters				
	ML	AP	MAP	LAP	AR
***This study***	77.52 ± 3.22(M)	49.26 ± 2.49(M)	51.79 ± 2.64(M)	46.73 ± 2.73(M)	1.57 ± 0.07(M)
	67.51 ± 3.17(F)	43.45 ± 2.53(F)	46.65 ± 2.44(F)	40.45 ± 2.37(F)	1.55 ± 0.09(F)
***Indians***					
Bansal et al. [1] Reddy et al. [33]	72.02 ± 5.13(M)*	47.80 ± 3.65(M)	48.58 ± 4.41(M)*	45.54 ± 1.00(M)*	
	67.58 ± 5.69(F)	43.53 ± 3.40(F)	43.19 ± 4.74(F)*	42.97 ± 2.92(F)*	
	74.70 ± 3.60(M)*	48.90 ± 2.60(M)			
	65.80 ± 3.90(F)*	43.10 ± 3.00(F)			
***Koreans***					
Kwak et al. [23]	76.10 ± 4.00(M)*	48.20 ± 3.30(M)*	48.50 ± 3.70(M)*	44.60 ± 3.20(M)*	1.58(M)
	67.64 ± 3.12(F)	43.20 ± 2.30(F)	43.50 ± 2.90(F)*	39.80 ± 2.50(F)	1.56(F)
***Americans***					
Mensch et al. [30]	80.30 ± 3.70(M)*		54.30 ± 3.60 (M)*	43.50 ± 2.80 (M)*	
	70.10 ± 2.80(F)*		46.00 ± 2.10 (F)	38.30 ± 2.60 (F)*	
***Caucasians***					
Dai et al. [9]	78.13 ± 3.91(M)		54.35 ± 2.99(M)*	48.62 ± 2.95(M)*	1.12 ± 0.05(M)*
	69.11 ± 2.82(F)		47.95 ± 2.36(F)*	42.63 ± 2.31(F)*	1.13 ± 0.05(F)*
***Japanese***					
Uehara et al. [37] Miyatake et al. [31]	77.90 ± 4.10(M)	54.10 ± 3.00(M)*			
	69.50 ± 3.40(F)*	49.20 ± 2.90(F)*	51.00 ± 2.60(M)*	46.10 ± 2.40(M)	
	76.40 ± 3.20(M)*		46.00 ± 2.70(F)*	41.30 ± 2.20(F)*	
	68.30 ± 2.90(F)*				
***Chinese***					
Cheng et al. [8] Yue et al. [39]	76.40 ± 2.80(M)*	51.30 ± 2.00(M)*	53.30 ± 2.50(M)*	47.70 ± 2.70(M)*	1.49 ± 5.70(M)
	68.80 ± 4.60(F)*	45.70 ± 1.90(F)*	47.50 ± 2.40(F)*	42.40 ± 2.30(F)*	1.51 ± 6.10(F)
	75.20 ± 3.60(M)*	41.50 ± 2.10(M)*	46.10 ± 2.10(M)*	36.80 ± 2.10(M)*	1.82 ± 0.07(M)*
	66.20 ± 2.10(F)	37.30 ± 2.80(F)*	41.50 ± 3.00(F)*	33.20 ± 3.20(F)*	1.78 ± 0.10(F)*
***Iranians***					
Karimi et al. [21]	77.80 ± 3.78(M)	48.79 ± 3.08(M)	53.14 ± 3.21(M)*	51.94 ± 3.57(M)*	1.60 ± 0.10(M)*
	66.52 ± 4.48(F)	43.07 ± 2.68(F)	45.48 ± 2.98(F)*	43.71 ± 3.46(F)*	1.55 ± 0.11(F)
***Turkish***					
Erkocak et al. [10]	77.10 ± 5.10(M)	47.60 ± 3.80(M)*	53.90 ± 4.20(M)*	45.90 ± 3.70(M)	1.62 (M)*
	68.70 ± 3.60(F)*	40.90 ± 3.10(F)*	47.50 ± 3.90(F)*	39.90 ± 3.30(F)	1.68 (F)*

*Statistical significance *p* < 0.05

Besides, the MAR and LAR of proximal tibias were determined and compared with other studies. The measurement results are shown in Table 7. Compared to the other studies in Caucasians, Indians, and Japanese, the MAR and LAR were more petite, especially on the medial side. However, the LAR was similar to Japanese for males and Indians and Caucasians for females.

**Table 7:** Comparison of the anterior radius of proximal tibias with other studies in mean ± SD (mm)

Studies	Population	MAR		LAR	
		Male	Female	Male	Female
This study	Thais	26.00 ± 1.41	22.86 ± 1.43	23.00 ± 1.38	20.03 ± 1.29
Bansal et al. [1]	Indians	29.95 ± 2.87*	26.74 ± 3.17*	22.21 ± 3.31*	19.99 ± 3.57
Dai et al. [9]	Caucasians	38.67 ± 7.61*	34.39 ± 6.40*	24.16 ± 4.22*	20.47 ± 3.77
	Indians	39.32 ± 9.57*	32.92 ± 8.37*	24.22 ± 4.07*	18.43 ± 3.52*
	Japanese	35.23 ± 7.57*	29.59 ± 4.80*	22.50 ± 2.59	18.74 ± 2.50*

*Statistical significance *p* < 0.05

### Comparison to the current tibial implants

**Fig. 4:**
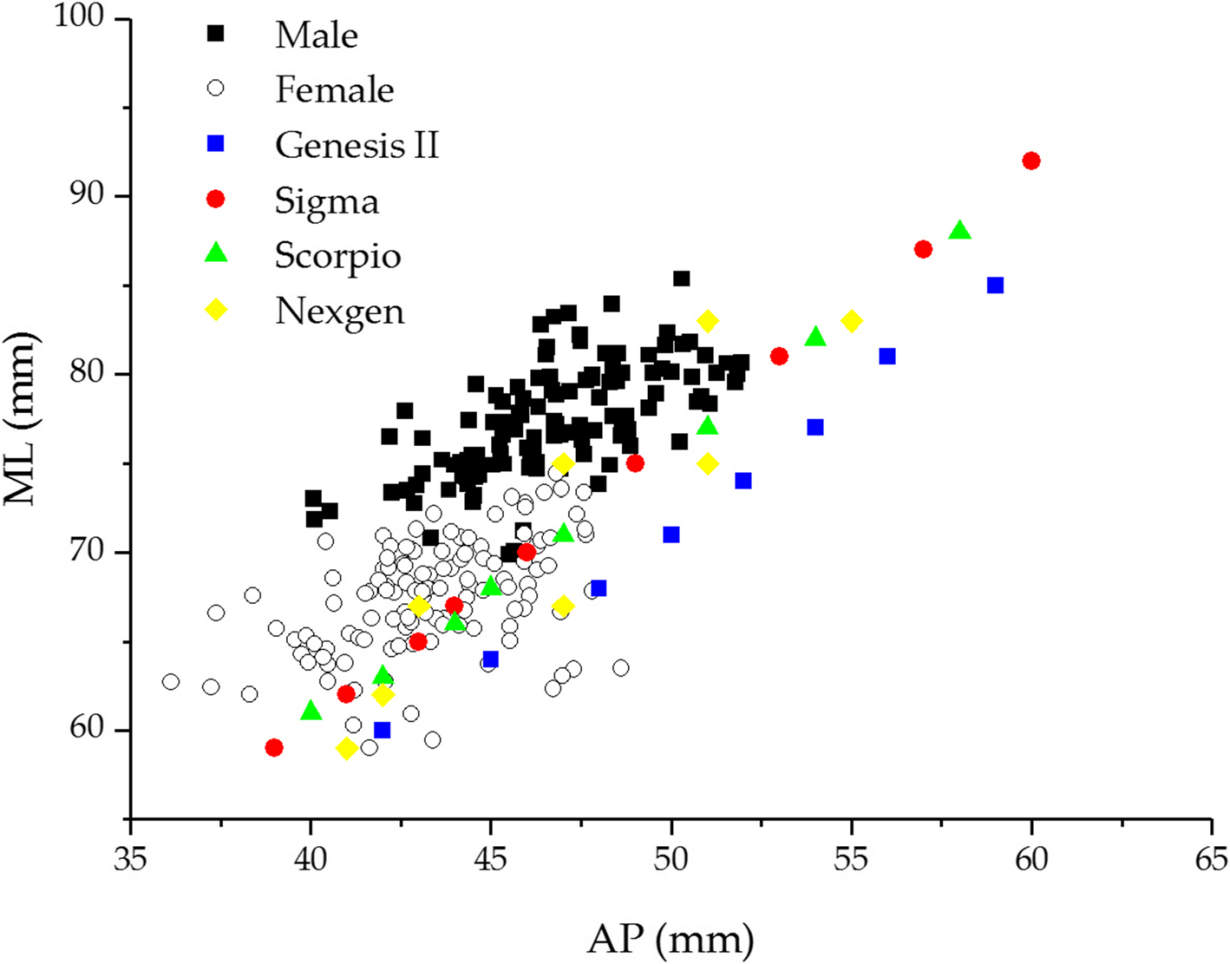
The relationship of anteroposterior width (AP) and mediolateral length (ML) compare between Thai proximal tibia data and four commercial tibial components

Generally, the ML and AP are used to select the implant size and identify the ideal size of the tibial component required. Therefore, these parameters were compared to the dimension of the commercial implant commonly used in Thailand. In this study, the typical commercial systems, including the ZIMMER (Nexgen), DEPUY (Sigma), STRYKER (Scorpio), and SMITH & NEPHEW (Genesis II), were used and displayed in a scatter diagram. Figure 4 depicts the relationship between AP and ML of the tibial implant and Thai tibia dimensions in males and females. The results revealed a mismatch between Thai tibial and commercial component dimensions, especially in the lack of coverage of the ML dimension. The AP dimension of typical implants tended not to be suitable to Thai anatomy with high mediolateral overhang. In addition, this finding showed that Thai females required smaller implant sizes with the same AP, while Thai males required a more significant ML than females. Although the size number of commercial implants covered the Thai tibia, the sizes of all implants did not match the Thai tibia. As a result, the tibial component should be improved to be suitable for the Thai population, for example, the decreasing of the AP dimension and increasing the number of the ML dimension in the same AP such as the Nexgen system.

**Fig. 5:**
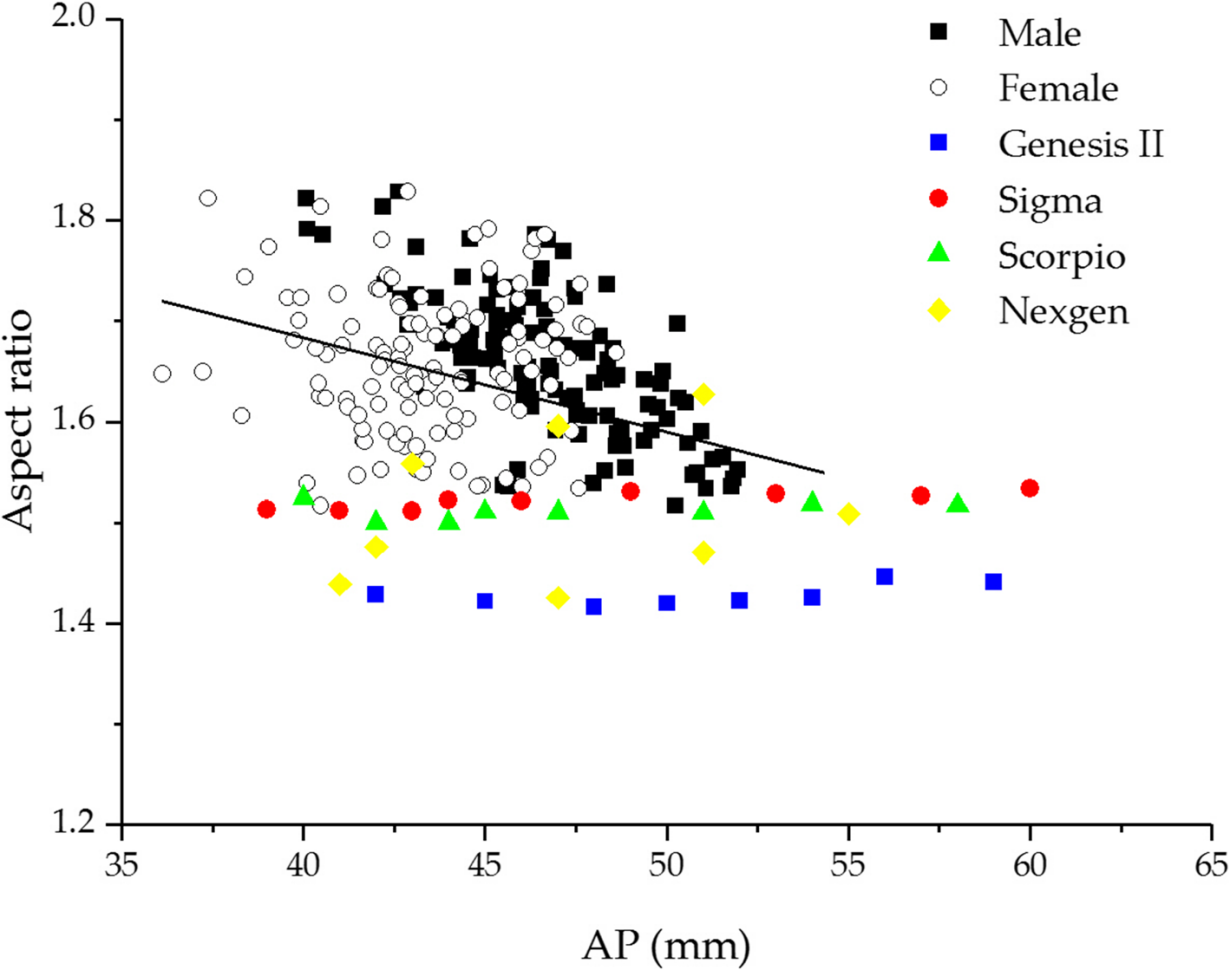
Tibial aspect ratio (AR) and anteroposterior width (AP) of Thai knee compared with four tibial components

To determine whether the tibial component of total knee arthroplasty would be appropriate for the Thai population or not, the tibial aspect ratio was calculated and compared to four different implants. Figure 5 shows the relationship between aspect ratio and AP. The results revealed that the aspect ratio had a definite negative correlation with the increasing AP. The aspect ratio of Thai males was more significant than Thai females with the same AP. Only the ZIMMER (Nexgen) system displayed a changing aspect ratio compared to the four implants. However, it was increasing, whereas the aspect ratio of the Thai tibia was decreasing with the increasing AP. Furthermore, the aspect ratio of the other implants showed a relatively constant ratio.

## Discussion

In this study, the morphometric data of the Thai proximal tibia was assessed using 3D models. Reverse engineering techniques were used to reconstruct these models from CT data. Based on the 3D measurements, it provided greater convenience and accuracy than alternative methods [11]. The proximal tibia morphometric parameters are utilized for various purposes, including sex determination, ethnic identification, and primary data to design the total knee prosthesis [1, 6, 8, 15, 37, 39].

According to the findings of this study, the MAP has higher values than LAP, consistent with other reports [14, 23, 30]. In addition, the MAR was more significant than the LAR. The CM and CL did not have equal dimensions, with the CM being small. These findings could imply that an asymmetric prosthesis design could improve coverage between the implants and the resected surface of the tibia. Alternatively, several authors have indicated that the bone coverage of symmetric design can be improved [17]. Further study is needed to determine whether an asymmetric design is better or not. Furthermore, the dimensions of the right and left sides were not significantly different. When comparing male and female proximal tibia parameters, all the male parameters were significantly higher than females, as discovered by previous studies [5, 8, 23, 24, 30, 37]. However, the males and females had slightly different aspect ratios. Thais were clearly determined to have a range of the tibial aspect ratio between 1.55 to 1.57. In addition, the anterior radius was measured on both the medial and lateral plateaus of the tibia, which to our knowledge, has not ever been reported in Thai morphometric studies. The proximal tibia parameters in this study revealed that ML, AP, and AR were slightly higher than previously reported studies concerning anthropometry of the knee joint in Thais measured based on MRI images [5]. These differences may be caused by different parameter definitions and methods of measurement. For example, this study performed measurements at the level of tibial resection 6 mm below the lateral tibial plateau while other studies [5] measured at the level of tibial resection 10 mm.

The morphometric data on the proximal tibia in males and females were analyzed using linear regression and correlation analysis as presented in Table 5 to assess the relationship between each parameter. Between specific parameters, there was a positive correlation. ML and LAP possessed the highest correlation coefficients (0.859). Furthermore, the correlation coefficients between ML and MAP, ML and AP were 0.818 and 0.811, respectively, a fact also reported in Korean studies [23]. The high correlation (between 0.7 to 1.0) indicates a highly linear relationship between the pairwise parameters. The linear regression equation was considered to estimate the dependent variable precisely, such as predicting other morphometric parameters.

Asian knees are smaller than Caucasian knees, according to numerous studies [8, 23, 37]. According to the present study, Koreans, Japanese, Chinese, Iranians, and Turkish had a similar ML and AP, including the medial and lateral sides. However, several parameters differed somewhat from this study, such as slightly higher AR in Chinese [39]. This difference could be due to their bone resection technique compared to this study [23]. Besides, the values of morphometric parameters were slightly higher in comparison to Indians. However, ML, MAP, and LAP were smaller than Americans, clearly evidenced in males. It can be seen that the morphometric of the proximal tibia differs between nationalities. As a result, knee prostheses designed using Western anatomy may not suit other races, particularly Asians. Therefore, the design of knee prostheses for Thais should be improved to suitably accommodate the Thai knee.

The results revealed a mismatch between the Thai tibia and the commonly available tibial component. Figure 4 depicted the correlation between the tibial component and proximal tibia of Thais. The findings revealed a relative correlation with Sixma, Nexgen, and Scorpio but a low correlation with Genesis II, which may be caused by a low aspect ratio by design. This aspect ratio may be appropriate for Westerns and Caucasians. All prostheses systems were undersized in ML with the smaller AP size and overhung in ML with the larger AP size, a fact especially noticeable in the male group. Regardless of gender, the tibial aspect ratio had a consistent negative correlation with increasing AP dimension. Compared to the tibial component, the Nexgen showed a positive correlation in aspect ratio change, whereas the others showed a relatively constant ratio. The constant aspect ratio of the knee prosthesis did not correspond to the anatomy of the Thai tibia. The mismatches between implant dimensions and bone caused an overhang or underhang of the tibial component. The tibial overhanging may cause soft tissue irritation and knee pain, while the underhanging of the tibial component has the potential to increase tibial bone resorption, which is one of the causes of aseptic loosening [3, 12, 26]. As a result, Thai-specific knee prostheses are required.

**Fig. 6:**
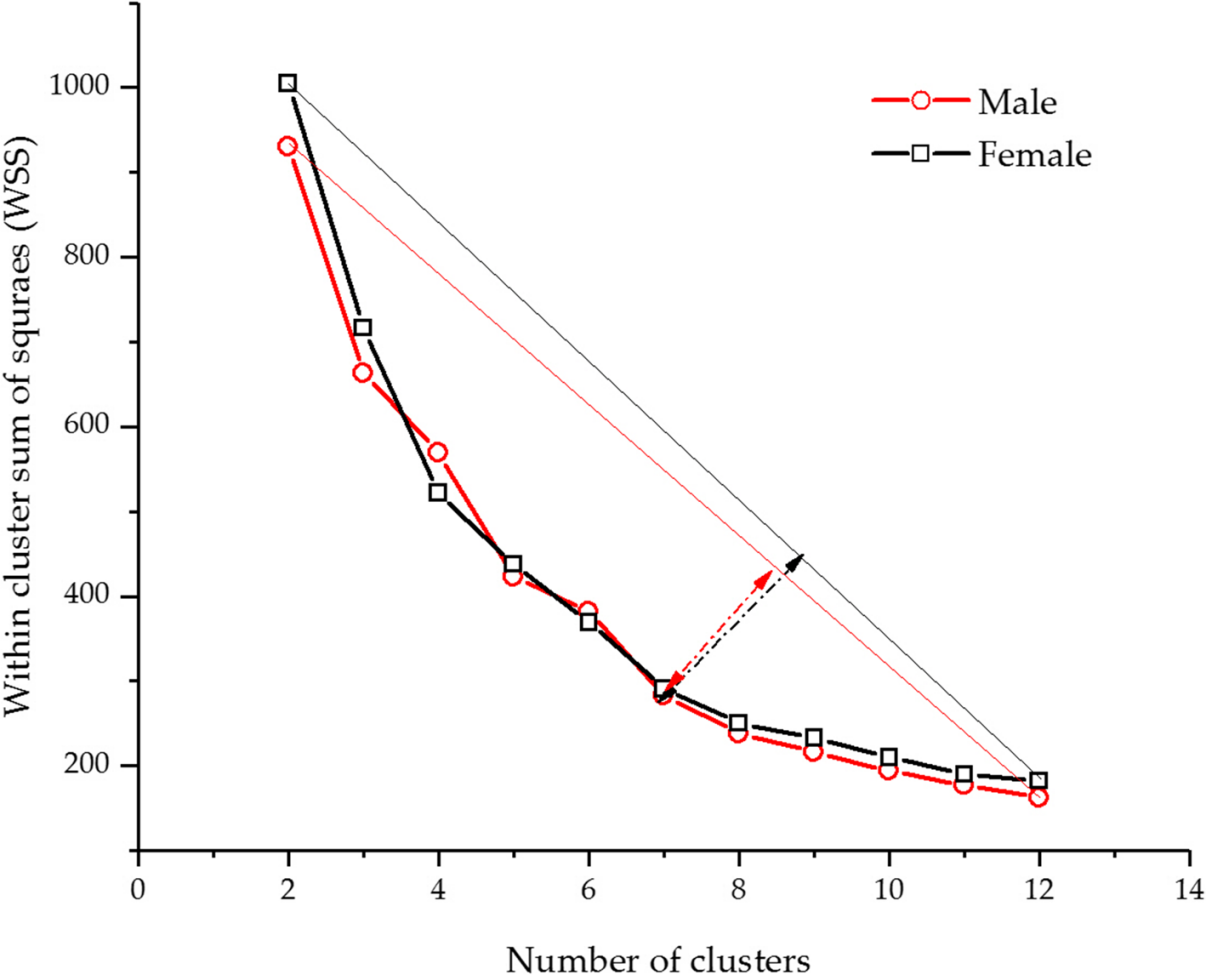
The relationship between the total within-cluster sum of squares (WSS) and cluster numbers

K-means clustering analysis was used to find the optimum number of tibial component sizes. Figure 4 shows the correlation between the ML and AP of the proximal tibia. This correlation was used to find the number of tibial component sizes which covered Thai knees. The number of clusters ranged from two to twelve for clustering analysis. The cluster centroid and total within-cluster sum of squares (WSS) were calculated for each cluster. The correlation between WSS and cluster numbers was plotted as curves to find the optimal cluster numbers, as shown in Fig. 6.

A bending point location in the graph is usually used to estimate the number of appropriate clusters [36]. When the bending point was not clearly visible, the longest distance between a straight line connecting the first and last points of clusters and any point of cluster indicated the optimal number of clusters. In Fig. 6, the result revealed seven sizes of the tibial component, the optimum number for coverage of the Thai tibia. The centroid data for each group represented the ML and AP dimensions of the tibial component for Thais. Table 8 shows the number of sizes and dimensions of tibial components for Thais.

**Table 8:** The recommended tibial component size for the Thais

Size	Male		Female	
	AP (mm)	ML (mm)	AP (mm)	ML (mm)
1	42	72	38	62
2	44	75	40	65
3	46	72	42	62
4	46	78	43	68
5	48	75	44	68
6	50	78	46	71
7	52	80	48	65

**Fig. 7:**
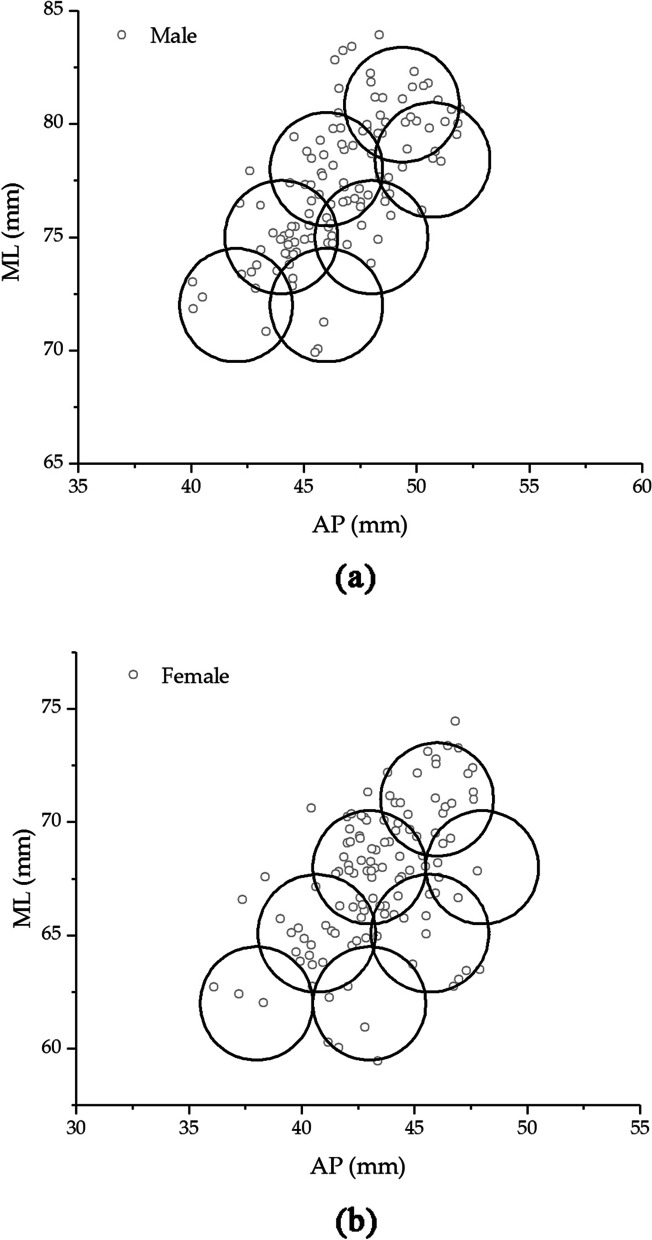
The percentages of the tibial component coverage on tibial cut surface related to AP and ML dimensions. **a** males and **b** females

The newly designed tibial components (seven sizes) were plotted as a circle, with each size of AP and ML dimensions as the center of the circle. The circle’s radius of 2.5 mm represented a range of criteria for underhang or overhang of the implant [7]. The absolute overhang was defined as more than 2 mm, while the relative overhang was defined as less than 2 mm. Absolute underhang was defined as under cover of more than 5 mm, while relative underhang was determined under 2 to 5 mm [20]. Figure 7 depicted the coverage percentages between the tibial component and the tibial cut surface according to AP and ML dimensions. The findings revealed that females had coverage percentages of around 96%, while males had coverage percentages of 95%. However, the tibial components in this study were symmetrical designs. The medial plateau is more extensive than the lateral plateau; thus, the asymmetry design should also be considered.

In this study, there were some limitations. Firstly, the volunteers were healthy, not TKA candidates. The morphometric dimension of the normal knee may differ from that of the osteoarthritic knee [8]. Secondly, most volunteers lived in the Northeast of Thailand. The distinction in residence may affect the average values of proximal tibia morphology in Thais [34]. Thirdly, while this study used 3D models to measure morphometric parameters, the effect from cartilage thickness was not included. These models were reconstructed using CT images, depending on the threshold value and mesh quality. Finally, this study only measured one resected surface in the tibia, but the actual surgery determines the depth of the cut surface depending on the stage of disease [23]. Despite these limitations, the current study provides relevant and necessary morphometric data on Thai tibias.

## Conclusion

The morphometric parameters of the Thai proximal tibia were not significantly different between the right and left sides. When comparing with gender, all male parameters were significantly higher than females. The majority of the pairwise parameters had a strong positive correlation. There was a significant difference in morphometric dimensions between nationalities, with Thais having smaller proximal tibias than Caucasians. There was also a mismatch between the Thai proximal tibia and the tibial component of TKA, which is generally used. The design of the tibial component should be recommended to cover the anatomy of the Thai knee, especially in the ML and AP sizes. This study provides essential information for the development of Thai-specific knee prostheses.
